# Application of Monopotassium Dipropylene Glycoxide for Homopolymerization and Copolymerization of Monosubstituted Oxiranes: Characterization of Synthesized Macrodiols by MALDI-TOF Mass Spectrometry

**DOI:** 10.3390/polym12122795

**Published:** 2020-11-26

**Authors:** Zbigniew Grobelny, Justyna Jurek-Suliga, Sylwia Golba

**Affiliations:** 1 Institute of Chemistry, University of Silesia, 40-007 Katowice, Poland; zbigniew.grobelny@us.edu.pl; 2 Institute Materials Engineering, University of Silesia, 40-007 Katowice, Poland; sylwia.golba@us.edu.pl

**Keywords:** oxiranes, homopolymerization, copolymerization, macrodiols, MALDI-TOF

## Abstract

Monopotassium dipropylene glycoxide, activated by a 18-crown-6 cation complexing agent (K-DPG/L, where DPG (dipropylene glycol) is a mixture of isomers) was used as an effective initiator of the homopolymerization and copolymerization of several monosubstituted oxiranes, i.e., propylene oxide (PO), 1.2-butylene oxide (BO), and some glycidyl ethers such as allyl, isopropyl, phenyl, and benzyl ones (AGE, IPGE, PGE, and BGE, respectively). The copolymers are novel and can be prospectively used for the fabrication of new thermoplastic or crosslinked polyurethanes. All processes were carried out in homogeneous mild conditions, i.e., tetrahydrofuran solution at room temperature and normal pressure. They resulted in new unimodal macrodiols with M_n_ = M_calc_ in the range of 1500–8300, low dispersity M_w_/M_n_ = 1.08–1.18 and a chemical structure well defined by several techniques, i.e., MALDI-TOF, size exclusion chromatography (SEC), ^13^C NMR, and FTIR. Monopotassium salts of homopolyether-diols, i.e., PPO-diol, PBO-diol, and PAGE-diol, appeared to be useful macroinitiators for the preparation of new triblock copolyether-diols by polymerization of glycidyl ethers. In BO/BGE random copolymerization initiated with K-DPG/L, macromolecules of copolyether-diol were exclusively formed. Macromolecules of copolyether-diol accompanied by homopolyether PPO-diol were identified in the PO/PGE system. However, AGE and PGE reacted by giving random copolyether-diol as well as homopolymer-diols, i.e., PAGE-diol and PPGE-diol. Macromolecules of prepared copolyether-diols contain various numbers of mers deriving from comonomers; the kind of comonomer determines the composition of the product. Several prepared homopolyether-diols and copolyether-diols could be useful for the synthesis of new thermoplastic polyurethanes.

## 1. Introduction

Polyether-polyols are the most important macromonomers for the synthesis of polyurethane elastomers and crosslinked foams [1,2,3,4,5,6,7,8]. They are usually prepared via neat anionic ring-opening polymerization of ethylene oxide (EO), propylene oxide (PO), or 1,2-butylene oxide (BO) using KOH (catalyst) and 1,2-propylene glycol or glycerol (starters) at a high temperature (>100 °C) and pressure. The molar masses (M_n_) of polyether-diols and polyether-triols fabricated industrially from PO are usually 2000–4000 and 3000–6500, respectively [1]. The application of several novel initiators for PO anionic polymerization, i.e., dipotassium salts of resorcinol, 4,4′-bisphenol, or 4,4′-sulfonyldiphenol, gave polyether-diols with relatively low M_n_ = 1000–3000 [9]. Recently [10], we used for initiation dipotassium salts of various aliphatic glycols activated by coronand 18-crown-6 (18C6). This allowed us to prepare bimodal PPOs containing a fraction with two terminal OK groups (M_n_ = 10,000, ~30% yield) and fraction with unsaturated allyloxy and OK terminal groups (M_n_ = 30,000, ~70% yield). Both kinds of terminal groups can be easily converted to OH ones [2]. Higher unsaturation (~83%) was observed in PPOs obtained in the presence of a suspension of anhydrous KOH in tetrahydrofuran (THF) [11]. Moreover, cyclic oligo(potassium glycidoxide)s with three or six OK groups of activated 18C6 were applied as macroinitiators for the polymerization of PO, BO, and styrene oxide (SO) [12]. Star-shaped polyether-polyols with M_n_ = 1200–8000 were prepared in this way. Similar products were recently obtained by the polymerization of PO [13] and SO [14] initiated with potassium salts of 2,2,6,6—tetrakis(hydroxymethyl)cyclohexanol. These polymers can be used as substrates for the synthesis of new crosslinked polyurethanes [13].

The most popular classes of polyether-diols are PPO-diols, block copolymers PO-EO with terminal PEO blocks, block copolymers PO-EO with internal PEO blocks, and random PO-EO copolymers [1]. They are used especially for polyurethane elastomers, coatings, adhesives, and sealants [15,16]. The most extensively studied triblock copolyether-diol is PEO/PPO/PEO, a polymeric nonionic surfactant, which is commercially available as Pluronics (BASF) or Symperonics (ICI) [15,16,17]. In this polymer, PPO builds the hydrophobic block, whereas PEO has two hydrophilic blocks. Then, a number of ABA and BAB triblock copolymers of ethoxyethyl glycidyl ether (EEGE) and PO were prepared by sequential anionic polymerization initiated with dicesium salt of dipropylene glycol [17]. It is known that these copolymers can form nanosized aggregates and exhibit different temperature behavior depending on the copolymer architecture.

In the synthesis of polyether-diols, the most frequently oxirane used is propylene oxide. The literature data concerning application of other monosubstituted oxiranes are rather scarce. Stolarzewicz at al. [18] studied the polymerization of chlorophenyl glycidyl ethers in the presence of powdered KOH and proposed that the process was initiated both in solution and on the surface of the initiator. A similar effect was also observed in the polymerization of PO [19], BO, SO, and various glycidyl ethers mediated with a suspension of anhydrous KOH in THF [20]. The molar mass, dispersity, and modality of synthesized polyether-diols depend strongly on the initial concentration of reagents, their solubility in the reaction mixture, and the presence of water or cation complexing agents, i.e., coronand 18C6 and cryptand C222. Recently, we applied another type of initiator (hydroxyalkoxide), i.e., monopotassium dipropylene glycoxide activated by 18C6 for homogenous polymerization of PO [10] and SO [14], as well as copolymerization of the latter with PO and glycidyl ethers [21]. This initiator contains two active sites in the molecule, resulting in polyether-diols and a very low number of macromolecules with unsaturated end groups, reduced by the presence of OH end groups in polymer chains. Thus, this initiator seemed to be much more useful than KOH in the synthesis of polyether-diols from monosubstituted oxiranes. It is active even without the addition of crown ether, but this addition increased their activity due to the formation of crown separated ion pairs. It results in increase of polymerization rate and yield of product.

In the present work, we applied monopotassium dipropylene glycoxide activated by 18C6 for the preparation of several new homopolyether-diols and copolyether-diols, i.e., triblock or random ones. PO, BO, isopropyl glycidyl ether (IPGE), allyl glycidyl ether (AGE), phenyl glycidyl ether (PGE), and benzyl glycidyl ether (BGE) were used as monomers. These copolymers are novel and bring innovation to the work. All processes were performed in mild conditions, i.e., in THF solution at room temperature. Characterization of the polymers was made by SEC, MALDI-TOF MS, and supporting techniques, such as ^13^C NMR and FTIR. The main analytical tool was MALDI-TOF MS, which enables insight into the composition of the polymers and thorough characterization of the obtained polymers. Synthesized materials could be potentially useful for the preparation of new elastomeric or crosslinked polyurethanes in reactions with diisocyjanates or poly(diisocyjanates), respectively.

## 2. Experimental

### 2.1. Materials

Monomers, i.e., propylene oxide, 1,2-butylene oxide, isopropyl glycidyl ether, allyl glycidyl ether, phenyl glycidyl ether, and benzyl glycidyl ether (all from Sigma-Aldrich, Poznan, Poland, ACS reagent), were dried over CaH_2_ and distilled at 306 K (33 °C), 336 K (63 °C), 414 K (131 °C), 427 K (154 °C), 518 K (245 °C), and 344 K (71 °C)/11 Torr, respectively. Anhydrous tetrahydrofuran (THF) (Acros Organics, Poznan, Poland, ACS reagent) was kept over CaH_2_ and distilled at 339 K (66 °C) prior to use. A 35 wt % dispersion of KH in mineral oil (Sigma-Aldrich, Poznan, Poland, ACS reagent) was mixed with *n*-pentane in a dry argon atmosphere and then decanted. This was repeated three times, followed by a three-fold washing with dry THF. Finally, THF was evaporated in a vacuum. The KH present was determined by a standard gas law calculation of the hydrogen liberated after treatment with 2-butanol (1.0 H_2_ = 1.0 KH). The resulting solution was titrated to a phenolphthalein end point. Very little excess (<1%) of total base over hydride base (from gas evolution) indicated the small hydrolysis of the original KH sample. Dipropylene glycol (mixture of isomers) (Sigma-Aldrich, Poznan, Poland, ACS reagent) and 18-crown-6 (18C6) (Merck, Warszawa, Poland, ACS reagent) were used without purification.

### 2.2. The Synthesis

All syntheses were carried out at room temperature in a 50 cm^3^ reactor equipped with a magnetic stirrer and a Teflon valve, enabling substrate delivery and sampling under an argon atmosphere.

#### 2.2.1. Homopolyether-Diols

A monopotassium salt of dipropylene glycol (K-DPG) was obtained through the reaction of potassium hydride with an appropriate amount of glycol dissolved in THF at 20 °C. The initial concentrations of the monomer were 2.0 or 5.0 mol/dm^3^ and the initial concentration of the initiator was 0.1 mol/dm^3^. For example, potassium hydride (0.08 g, 2.0 mmol) and THF (13 cm^3^) containing 18C6 (0.53 g, 2.0 mmol) were introduced into a 50 cm^3^ reactor equipped with a magnetic stirrer and a Teflon valve, enabling substrate delivery and sampling under an argon atmosphere. Then, dipropylene glycol (2.0 mmol) was added to the THF solution (0.5 mol/dm^3^, 4.0 cm^3^) by a syringe. The reaction mixture was stirred for 30 min until all hydrogen (44.7 cm^3^) was dissolved. This resulted in a solution of pure anhydrous monopotassium salt of dipropylene glycol activated ligand in THF. That system was used as the initiator, when propylene oxide (2.8 cm^3^, 2.3 g, 40.0 mmol) was introduced into the reactor. The reaction mixture was then stirred for several days. The course of polymerization was followed by assessing the changes in the content of oxirane groups in samples taken at various times from the reaction mixture. The content of oxirane groups was determined by the dioxane method [22]. A 0.2–0.7 g sample was taken from the reaction mixture and dissolved in 10 cm^3^ of a 0.2 mol/dm^3^ solution of HCl in 1,4-dioxane, the whole being then allowed to stand for 1 h at room temperature. The solution was then titrated with 0.1 mol/dm^3^ KOH using phenolphthalein as an indicator, with a blank determination being run simultaneously. Final conversion of the monomers (~99%) was calculated by NMR technique taking into account signals of oxirane groups in the spectrum. After complete conversion of the monomer, the reaction mixture was neutralized with a HCl/H_2_O system (0.1 mol/dm^3^, 70 cm^3^) and transferred to a separator containing chloroform (70 cm^3^). After shaking for 5 min, two layers formed, a bottom polyether layer and a top layer containing water and the potassium salt. These layers were separated and the top layer was removed. After three washes with distilled water, polyether was obtained by evaporating chloroform and water.

#### 2.2.2. Triblock Copolyether-Diols

Potassium hydride (0.08 g, 2.0 mmol) and tetrahydrofuran (12.5 cm^3^) containing 18C6 (0.53 g, 2.0 mmol) were introduced into the reactor and then a 0.5 mol/dm^3^ solution of dipropylene glycol in tetrahydrofuran (4.0 cm^3^) was added. The reaction mixture was stirred for 20 min until all the hydrogen (44.7 cm^3^) was evolved. This resulted in a solution of monopotassium salt of dipropylene glycol activated with a 18C6 complexing agent. That system was then used as the initiator when 1,2-butylene oxide (3.5 cm^3^, 0.42 g, 40 mmol) was introduced into the reactor. In this system, the initial concentration of the monomer was equal to 2.0 mol/dm^3^ and the initial concentration of the initiator was 0.1 mol/dm^3^. The reaction mixture was then stirred for several days. After complete conversion of the monomer, an equimolar amount of IPGE (4.7 cm^3^) was introduced into the reactor and stirred until total conversion of IPGE was achieved. Then the reaction mixture was neutralized with a HCl/H_2_O system (0.1 mol/dm^3^, 50 cm^3^) and transferred to a separator containing chloroform (70 cm^3^). After shaking for 5 min, two layers were obtained, a bottom copolyether layer and a top layer containing water and the potassium salt. These layers were separated and the top layer was removed. After three washes with distilled water, copolyether was obtained by evaporating chloroform and water in a vacuum. In subsequent experiments, various copolyethers were prepared in a similar way. The concentration of monomers during the polymerization was monitored by the NMR method. The final conversion was~99%, while the yields of the products were 97–99%.

#### 2.2.3. Random Copolyether-Diols

Potassium hydride (0.08 g, 2.0 mmol) and tetrahydrofuran (6.4 cm^3^) containing 18C6 (0.53 g, 2.0 mmol) were introduced into the reactor and then a 0.5 mol/dm^3^ solution of dipropylene glycol in tetrahydrofuran (4.0 cm^3^) was added. The reaction mixture was then stirred for 20 min until all the hydrogen (44.7 cm^3^) was evolved. This resulted in a solution of monopotassium salt of dipropylene glycol activated with a 18C6 complexing agent. That system was then used as the initiator, when a mixture of 1,2-butylene oxide (3.5 cm^3^, 0.42 g, 40 mmol) and equimolar amount of benzyl glycidyl ether (6.1 cm^3^) was introduced into the reactor and stirred until the complete conversion of monomers. Then, the reaction mixture was neutralized with a HCl/H_2_O system (0.1 mol/dm^3^, 50 cm^3^) and transferred to a separator containing chloroform (70 cm^3^). After shaking for 5 min, two layers were obtained, a bottom polymer layer and a top layer containing water and the potassium salt. These layers were separated and the top layer was removed. After three washes with distilled water, copolyether was obtained by evaporating chloroform and water in a vacuum. In subsequent experiments, other copolymers were prepared in a similar way. The final conversion was ~99% and the yields of the products were 97–99%.

### 2.3. Measurements

First, 100 MHz ^13^C nuclear magnetic resonance (NMR) spectra were recorded in CDCl_3_ at 25 °C on a BrukerAvance 400 pulsed spectrometer equipped with a 5 mm broadband probe and a Waltz16 decoupling sequence. Chemical shifts were referenced to tetramethylsilane, serving as an internal standard. To obtain a good spectrum of the polymer main chain exhibiting its microstructural details, about 3000 scans were satisfactory, but in order to observe the signals of the polymer chain ends more than 10,000 scans were necessary. Molar masses and dispersities of polymers were obtained by means of size exclusion chromatography (SEC) on a Shimadzu Prominence UFLC instrument at 40 °C on a Shodex 300 × 8 mm OHpac column, using tetrahydrofuran as a solvent. Poly(propylene glycol)s were used as calibration standards. Matrix-assisted laser desorption/ionization-time of flight (MALDI-TOF) spectra were recorded on a Shimadzu AXIMA Performance instrument. Dithranol was used as a matrix. Infrared (IR) spectra of samples were recorded on a Shimadzu IR Prestige spectrometer with an attenuated total reflectance (ATR) accessory. The diamond ATR crystal was purified prior to each measurement with isopropanol. Data were analyzed using the LabSolutions program. Each sample was scanned at a resolution of 2 cm^−1^.

## 3. Results and Discussion

### 3.1. Homopolyether-Diols

**Scheme 1:**
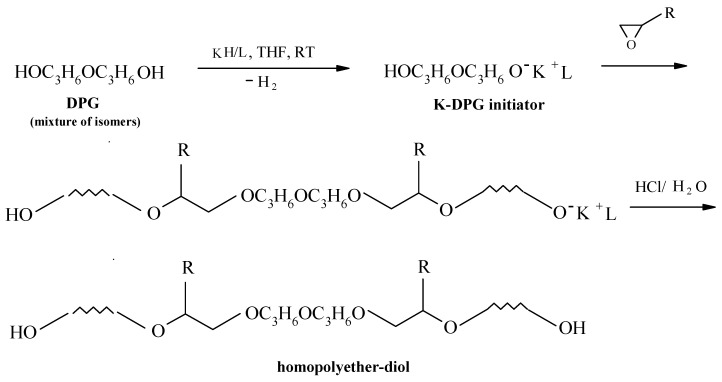
Preparation of initiator and homopolyether-diols by anionic ring-opening polymerization of monosubstituted oxiranes, where: R: CH_3_, CH_3_CH_2_, CH_2_=CHCH_2_OCH_2_, (CH_3_)_2_CHOCH_2_, PhOCH_2_ or PhCH_2_OCH_2_, L: 18C6.

Several macrodiols were synthesized from monosubstituted oxiranes in a homogeneous system at mild conditions according to the reaction shown in Scheme 1. The role of the 18C6 (L) ligand was the complexation of counterion in order to increase the reactivity of the initiator and the number of alkoxides in the growing polymer chain by the formation of crown ether separated ion pairs. This resulted in an increase in the initiator solubility, reaction rate, and yield of the product.

**Figure 1:**
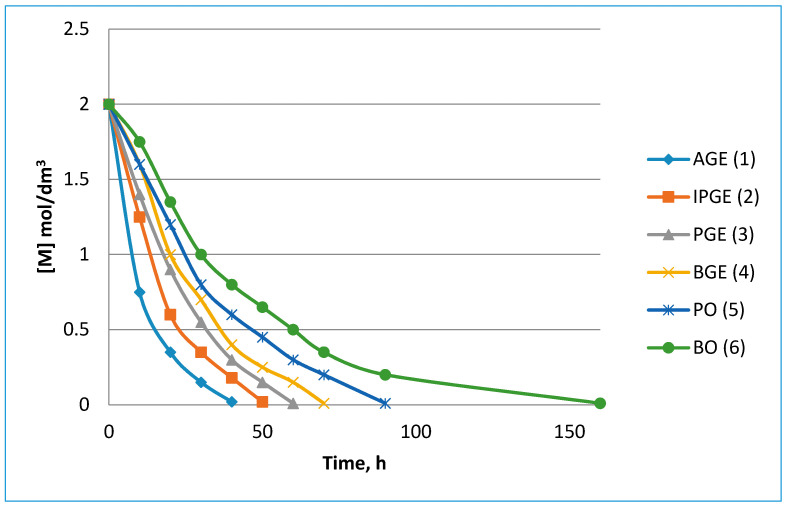
Monomer concentration vs. time of polymerization initiated with K-DPG activated by 18C6 in THF solution; [M]_o_ = 2.0 mol/dm^3^; [I]_o_ = [18C6]_o_ = 0.1 mol/dm^3^; AGE (**1**), IPGE (**2**), PGE (**3**), BGE (**4**), PO (**5**), and BO (**6**).

Conversion curves of the monomers are shown in Figure 1. The monomers were polymerized at various rates depending on the polar and steric effects of the substituent [23]. The reaction times were relatively long, which can result from intra-and/or intermolecular proton exchange between the OH and OK groups [21].

Table 1 shows data concerning the average molar masses (M_n_) and dispersities (M_w_/M_n_) of the mentioned polymers, as well as the initiator efficiency (*f* = M_calc_/M_n_) for complete conversion of the monomer. All polymers are unimodal and their dispersities are rather low (M_w_/M_n_ = 1.08–1.16).

**Scheme 2:**
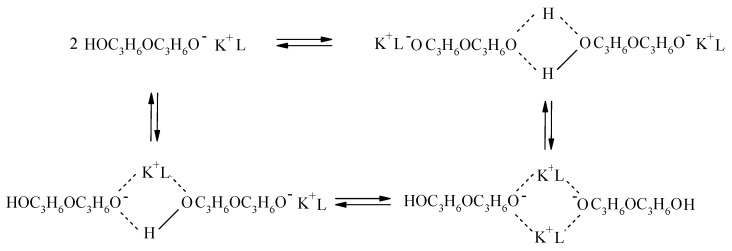
Equilibrium between two molecules of K-DPG/L and their ionic aggregates.

Molar masses (M_n_) of PPO-diols synthesized with K-DPG/L are higher than the calculated ones (M_calc_), indicating initiator efficiency *f* < 1. This effect can be explained by the strong tendency of the soluble initiator to the formation of various ionic aggregates existing in equilibrium (Scheme 2). Their reactivity varies, depending on the chemical structure and the polarity of the reaction mixture, which depends on the initial monomer concentration and possible monomer participation in the formation of the aggregates. These may influence on different initiator’s efficiency.

**Figure 2:**
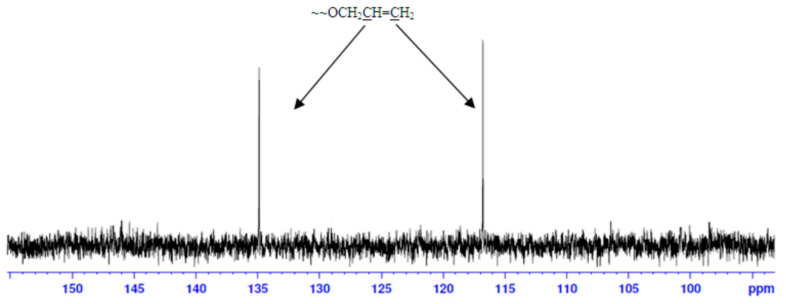
^13^C NMR spectrum of PPO-diol (1) showing signals of unsaturated allyloxy starting groups.

It was observed by the ^13^C NMR technique that the unsaturation of PPO-diols obtained with K-DPG is represented exclusively by allyloxy groups, CH_2_=CHCH_2_O– (116.71 ppm and 134.88 ppm, respectively), resulting from monomer deprotonation by –O^−^K^+^L groups of initiator and polymer chains (Figure 2). It is relatively low (1–4%) and increases with the initial monomer concentration. The presence of –OH end groups in macromolecules during the polymerization avoids the isomerization of allyloxy groups to *cis*-propenyloxy ones.

**Scheme 3:**
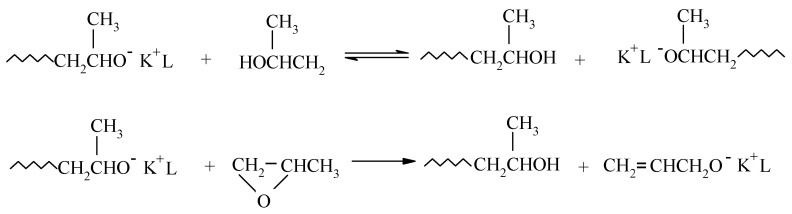
Cation exchange between potassium alkoxide and hydroxyl group and deprotonation of the CH_3_ group in the monomer.

In comparison, the unsaturation of polyethers was markedly higher when powdered anhydrous KOH was used as an initiator (~83%) at [M]_o_ and [I]_o_ equal to 2.0 and 0.1 mol/dm^3^ [11]. In the last case, mainly macromolecules containing isomeric *cis*-propenyloxy starting groups were formed. Moreover, the average molar mass of the polymer is much higher (M_n_ = 6000). The low unsaturation of the currently studied system results from the presence of OH groups in macromolecules. Evidently, the deprotonation of the latter by alkoxide end groups is faster than the deprotonation of the CH_3_ group in the monomer molecule, which markedly reduces the number of unsaturated groups (Scheme 3).

**Figure 3:**
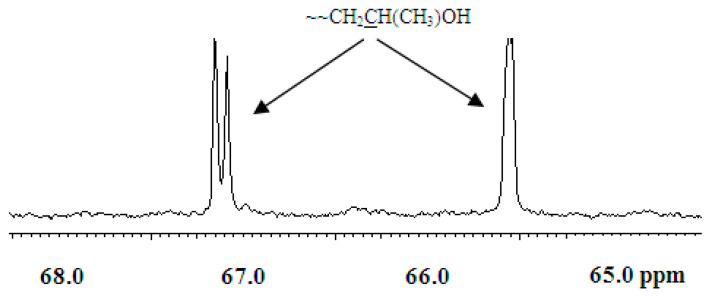
^13^C NMR spectrum showing the region of terminal groups in PPO-diol (1) prepared with K-DPG/L.

All PPO-diols (1–3) contain the same starting and ending –CH_2_CH(CH_3_)OH groups after quenching with HCl/H_2_O. For example, the ^13^C NMR spectrum of PPO-diol (1) revealed two signals of methine carbon bonded to the OH end group at 65.55 (65.54 and 65.56 ppm) and at about 67.1 ppm (67.09 and 67.15) ppm (Figure 3).

The splitting of the signal of the last units in the chain ends has already been discussed in our previous paper [11].

**Figure 4:**
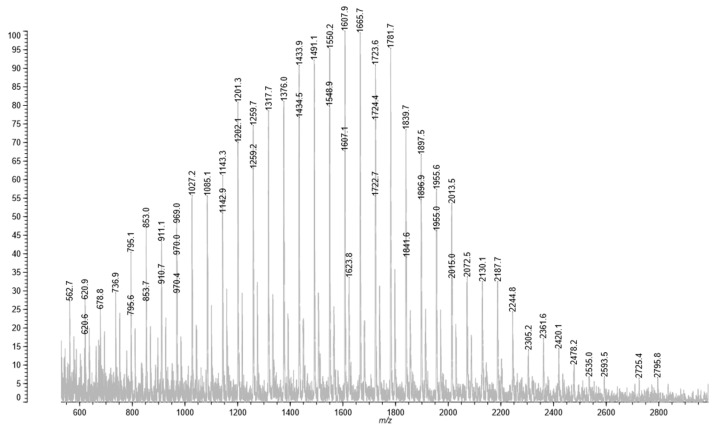
MALDI-TOF spectrum of PPO-diol (1) obtained with K-DPG/L.

MALDI-TOF analysis of the polymer confirmed its structure (Figure 4). Two series of signals were observed in the spectrum. The first one at m/z 620.9 to 2593.5 represents macromolecules possessing the central part (–OC_3_H_6_OC_3_H_6_O–) derived from the initiator and two terminal OH groups. For example, signals at m/z 736.9, 1317.7, and 2130.1 represent macromolecules containing 10, 20, and 30 mers of propylene oxide (M_calc_ = 738.0, 1318.8, and 2132.9, respectively). They exist as adducts with sodium ions. The second series at m/z 637.0 to 2609.6 represents macromolecules with the same structure forming adducts with potassium ions. For example, signals at m/z 1043.3, 1681.8, and 2436.2 belong to macromolecules with 15, 26, and 39 mers of propylene oxide (M_calc_ = 1044.5, 1683.3, and 2438.4, respectively). However, signals of macromolecules with allyloxy starting groups were not detected in the spectrum. It is worth noting that, in PBO-diols (4–6) and other polyether-diols, unsaturation was not observed using ^13^C NMR and MALDI-TOF techniques due to the effect of the substituent on the acidity of CH protons in the monomer molecule. In the ^13^C NMR spectrum of PBO-diol (4), a signal of the CH_3_ group of the initiator was observed at 17.10 ppm. The signals of the –CH_2_CH(CH_2_CH_3_)OH end group were at 73.46 and 73.70 ppm. However, signals at 65.55 and 67.10 pm derived from the initiator were not observed. This indicated that propagation occurred in two directions.

**Figure 5:**
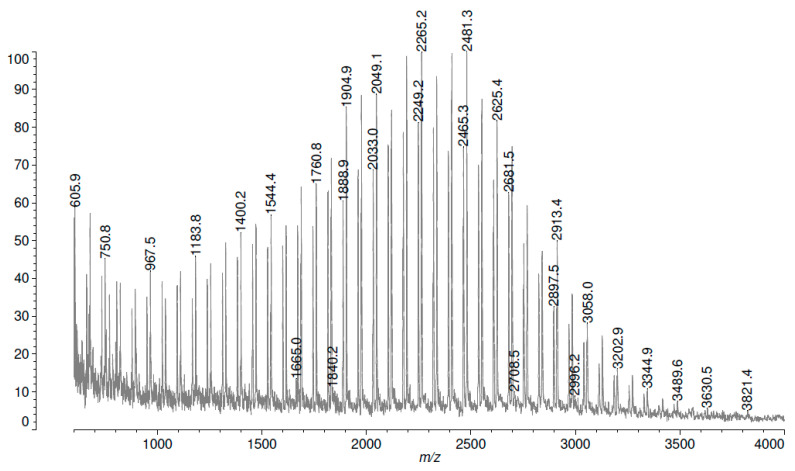
MALDI-TOF spectrum of PBO-diol (4) obtained with K-DPG/L.

In order to determine the chemical structure of PBO-diols, the MALDI-TOF technique was applied. The spectrum of the appropriate polymer is shown in Figure 5.

The spectrum in Figure 5 reveals two series of signals at m/z 600 to 4000. The first series at m/z 661.9 to 3762.7 represents macromolecules containing a central part derived from the initiator, several BO mers, and two end OH groups. For example, signals at m/z 1239.5, 2465.2, and 2897.3 represent macromolecules containing 15, 32, and 38 mers of BO (M_calc_ = 1238.8, 2464.7, and 2897.4, respectively). They formed adducts with sodium ions. The second series at m/z 605.9 to 3778.8 represents macromolecules with the same structure, which form adducts with potassium ions. For example, signals at m/z 750.8, 2049.1, and 2697.5 belong to macromolecules with 8, 26, and 35 mers of BO (M_calc_ = 750.2, 2048.1, and 2697.1, respectively).

**Figure 6:**
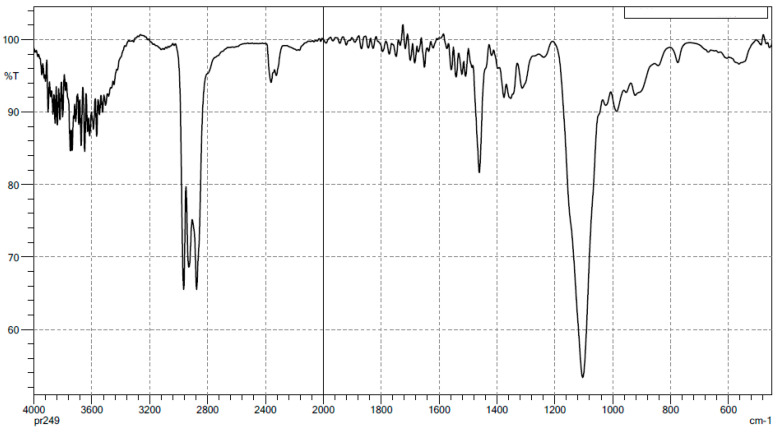
FTIR spectrum of PBO-diol (4).

For polyether-diols, FTIR ATR spectra were recorded to confirm their chemical structure based on selected mer units. The spectrum of PBO-diol (4) is shown in Figure 6.

Bands at 3000–2850 cm^−1^ reflect alkane-like fragments (–CH_2_–) of the main chains together with the bending/scissoring vibration of C–H in the range 1460–1440 cm^−1^ and rocking ones of C–H at 1370–1350 cm^−1^. For synthesized polyether bands, 3490 cm^−1^ represents the stretching vibration of associated –O–H, while the absorption peak at 1320 cm^−1^ is attributed to O–H bending vibration. Hydroxyl characteristic bands might also be identified at 1060 cm^−1^ for primary hydroxyl stretching [24]. For all polyethers, a strong C–O stretching band located in the 1200–1050 cm^−1^ wave number range is visible, but it is overlaid with an ether C–O–C band located at 1095 cm^−1^, characteristic of alkyl-substituted ether [25].

**Figure 7:**
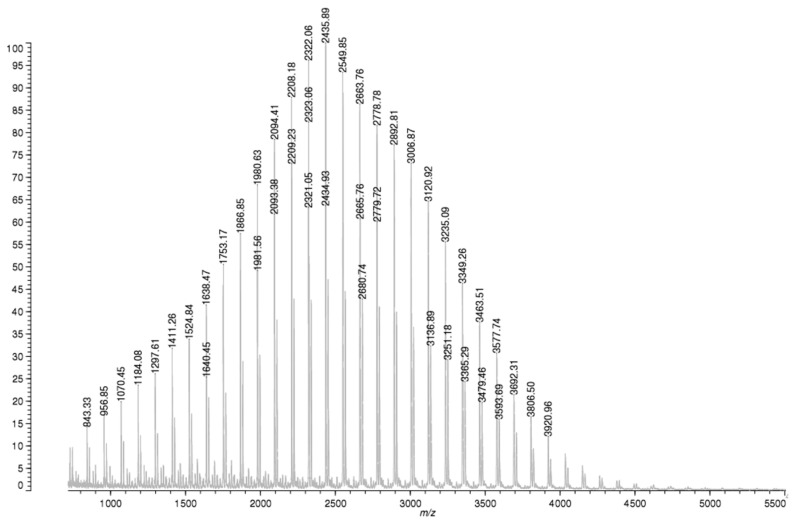
MALDI-TOF spectrum of PAGE-diol (7) prepared with K-DPG/L.

MALDI-TOF mass spectrometry was also used for the determination of the chemical structure of poly(glycidyl ether)s. The spectrum of PAGE-diol is presented in Figure 7.

Two series of signals were observed in the spectrum in Figure 7. The first one at m/z 727.9 to 4263.5 represents macromolecules possessing a central part derived from the initiator and two terminal OH groups. For example, signals at m/z 1070.4, 2667.3, and 3921.7 represent macromolecules containing 8, 22, and 33 mers of allyl glycidyl ether (M_calc_ = 1070.3, 2668.3, and 3923.8, respectively). They form adducts with sodium ions. The second series at m/z 744.0 to 4279.6 represents macromolecules with the same structure, which form adducts with potassium ions. For example, signals at m/z 1428.8, 3026.7, and 3595.5 belong to macromolecules with 11, 25, and 30 mers of the monomer (M_calc_ = 1428.8, 3026.8, and 3597.5, respectively).

**Figure 8:**
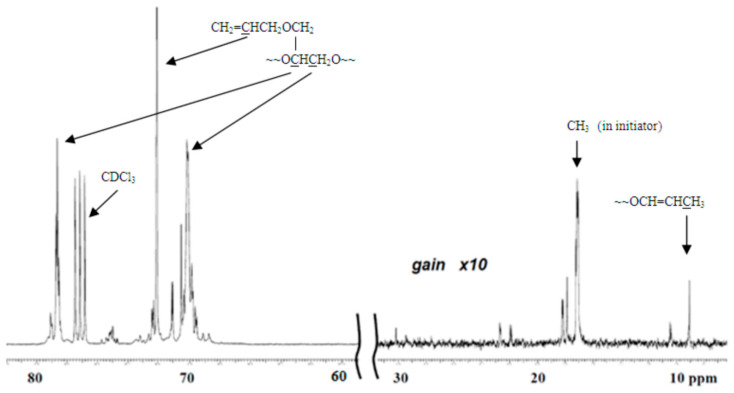
^13^C NMR spectrum of PAGE-diol (7) obtained with K-DPG.

Figure 8 presents the ^13^C NMR spectrum of PAGE-diol (7). The signal of the CH_3_ group of the initiator was observed at 17.10 ppm. The signals at 68.62 and 69.01 ppm can be attributed to the methine carbon of –OCH_2_CH(CH_2_OCH_2_CH=CH_2_)OH end group. It can also be stated that the alternative structure of the end group, i.e., –OCH(CH_2_OCH_2_CH=CH_2_)CH_2_OH, is highly improbable since in this case the signals of CH and CH_2_ carbons in the vicinity of the OH group should arrive at about 81.3 and 62.9 ppm, respectively, and there are no signals in these regions. This indicates regular chain propagation, and hence the opening of oxirane ring only in the β position.

It is also worth noting that the isomerization of allyloxy groups does not occur under the influence of –O^−^K^+^L active centers. The signals of *cis*-propenyloxy groups CH_3_CH=CHO previously observed at 9.2, 101.1 and 145.9 ppm in the polymerization of AGE initiated with anhydrous KOH (12%) [20] are very weak (0.1%). This is caused by the influence of the OH group derived from the initiator.

**Figure 9:**
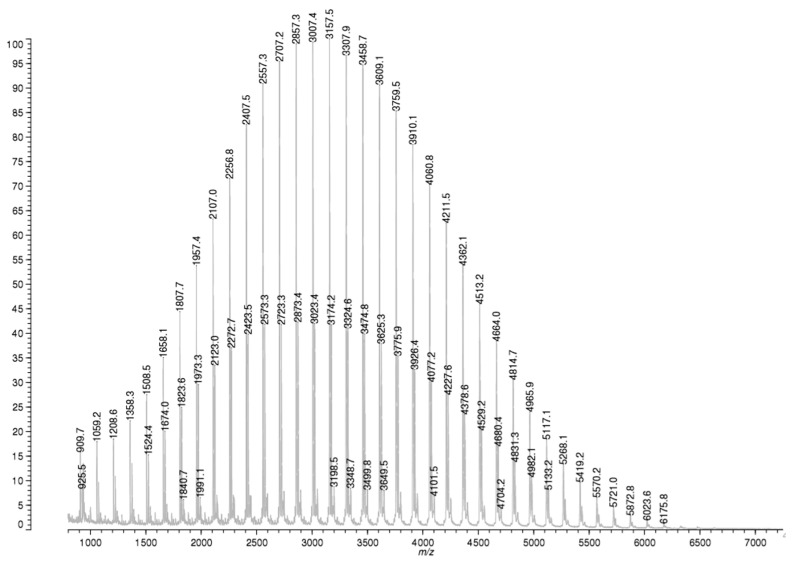
MALDI-TOF spectrum of PPGE-diol (11) synthesized with K-DPG/L.

Figure 9 shows the MALDI-TOF spectrum of PPGE-diol. Two series of signals were observed in the spectrum (Figure 9). The first series at m/z 924.0 to 5873.0 represents macromolecules possessing a central part derived from the initiator and two terminal OH groups. For example, signals at m/z 1225.3, 3176.1, and 4674.9 represent macromolecules containing 7, 20, and 30 mers of phenyl glycidyl ether (M_calc_ = 1224.5, 3176.7, and 4678.4, respectively). They form adducts with potassium ions. The second series at m/z 909.7 to 5856.9 represents macromolecules with the same structure, which form adducts with sodium ions. For example, signals at m/z 1959.6, 2710.1, and 4359.0 belong to macromolecules with 12, 17, and 28 mers of phenyl glycidyl ether (M_calc_ = 1959.2, 2710.0, and 4361.9, respectively).

**Scheme 4:**
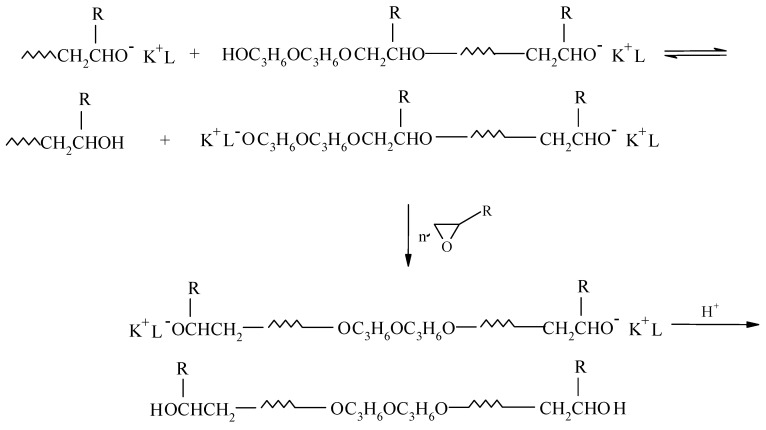
Formation of macromolecules with two active centers in growing chains.

Similar results were obtained for other polyether-diols synthesized in this work. This means that, during the polymerization, fast counterion exchange occurs between two macromolecules, which causes the propagation in two directions (Scheme 4).

When comparing the average molecular weight determined by MALDI-TOF and SEC, it can be seen that the results obtained by both methods are comparable but still show some discrepancies. For the samples with lower dispersities (M_w_/M_n_ < 1.10), the SEC-derived values are slightly lower (e.g., for sample 1: M_n_ = 1500 (SEC), M_n_ = 1600 (MALDI-TOF)), while for samples with higher dispersities (M_w_/M_n_ > 1.10), the SEC-derived values are slightly higher (e.g., for sample 7: M_n_ = 2700 (SEC), M_n_ = 2450 (MALDI-TOF)). This discrepancy is connected with the fact that the dispersity of the samples is a well-known factor that influences the MALDI-TOF spectra [26,27] and standards used for SEC calibration.

Summarizing, the application of potassium hydroxyalkoxide for the polymerization of monosubstituted oxiranes allows us to prepare several new linear polyether-polyols in mild conditions. Especially interesting for the synthesis of new polyurethane elastomers are macrodiols (Table 1, no 4–14) due to their unimodality and relatively high molar masses. The preparation of rigid crosslinked polyurethanes can also be possible in the reaction of macrodiols with poly(diisocyanates). They also appear to be useful as macroinitiators for the synthesis of new triblock copolyether-diols.

### 3.2. Triblock Copolyether-Diols

Several triblock copolyether-diols were synthesized using three macroinitiators based on PO(1), BO(4), and AGE(7). In each experiment, a macroinitiator was prepared by the ring-opening polymerization of the chosen monosubstituted oxirane (A) at [A]_o_ = 2.0 mol/dm^3^, initiated with monopotassium dipropylene glycoxide at [I]_o_ = 0.1 mol/dm^3^ in the presence of macrocyclic ligand 18-crown-6 (K-DPG/L). After complete conversion of the monomer, the second oxirane (B) was added to the reaction mixture and underwent quantitative copolymerization. Six triblock copolyether-diols were obtained after neutralization with HCl/H_2_O. The products were analyzed by several techniques. The SEC method indicated that all copolymers are unimodal. Their full characterization by this technique was shown in Table 2.

**Scheme 5:**
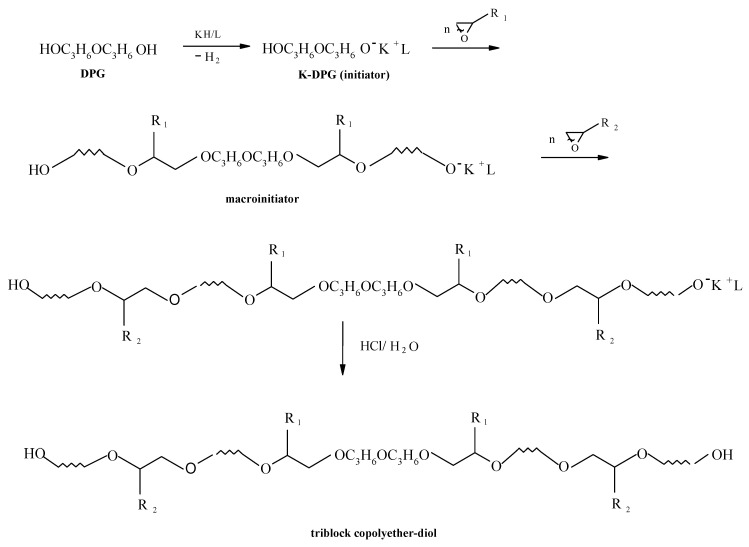
Preparation of triblock copolyether-diols by ring-opening polymerization of monosubstituted oxiranes, where: R_1_: CH_3_ and R_2_: CH_2_OCH_2_CH=CH_2_ or CH_2_OPh (for copolymer 15 or 16), R_1_: CH_2_CH_3_ and R_2_: CH_2_OCH_2_CH=CH_2_ or CH_2_OCH(CH_3_)_2_ (for copolymer 17 or 18), R_1_: CH_2_OCH_2_CH=CH_2_ and R_2_: CH_2_OCH(CH_3_)_2_ or CH_2_OCH_2_Ph (for copolymer 19 or 20).

The copolymerization course involving the synthesis of initiator and macroinitiator is shown in Scheme 5.

**Figure 10:**
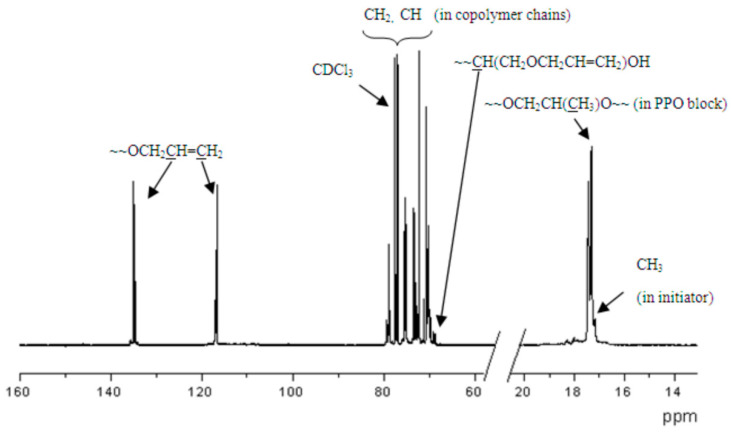
^13^C NMR spectrum of PAGE/PPO/PAGE triblock copolyether-diol (15).

In order to confirm the structure of macromolecules formed in the copolymerization, ^13^C NMR spectroscopy was applied. Figure 10 shows, for example, the spectrum of PAGE/PPO/PAGE copolymer (1). It reveals strong signals of carbons present in polymer chains, i.e., CH_2_ and CH (at 70–80 ppm), as well as substituents, i.e., CH_3_ (at 17.3 ppm) and OCH_2_CH=CH_2_ (at 116.6 and 134.9 ppm). The signal of terminal carbon of the CH(CH_2_OCH_2_CH=CH_2_)OH group was identified at 68.62 and 69.01 ppm. However, signals of terminal carbon of CH(CH_3_)OH group (at 65.55 and 67.10 ppm) were not found in the spectrum.

The lack of ^13^C NMR spectrum carbon signals of the end group derived from the macroinitiator indicated that, during the copolymerization, rapid cation exchange takes place. This allows polymer chains to grow in two directions. A similar phenomenon was observed in the copolymerization of other oxiranes.

**Figure 11:**
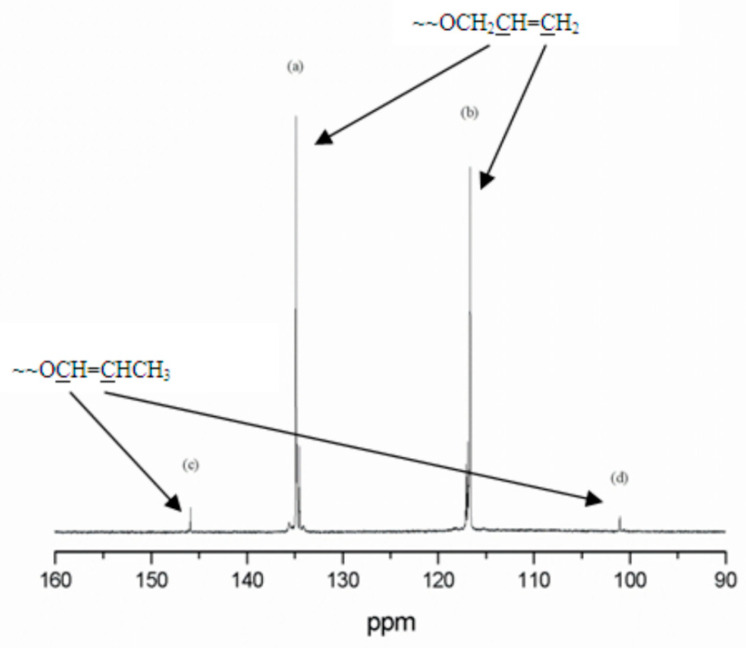
Signals of allyloxy (**a**,**b**) and *cis*-propenyloxy (**c**,**d**) groups observed in the unsaturated region of the ^13^C NMR spectrum of PAGE/PBO/PAGE copolyether-diol (17).

It is also worth noting that, for systems containing AGE as a comonomer, ^13^C NMR analysis of copolymers (Figure 11) indicated weak signals of *cis*-propenyloxy groups, i.e., OCH=CHCH_3_ (at 101.1 and 145.9 ppm). This resulted from the isomerization of allyl groups under the influence of active centers of growing polymer chains (Table 3). This effect was observed, before now, in the homopolymerization of AGE mediated with anhydrous KOH-activated cation complexing agents [19].

**Figure 12:**
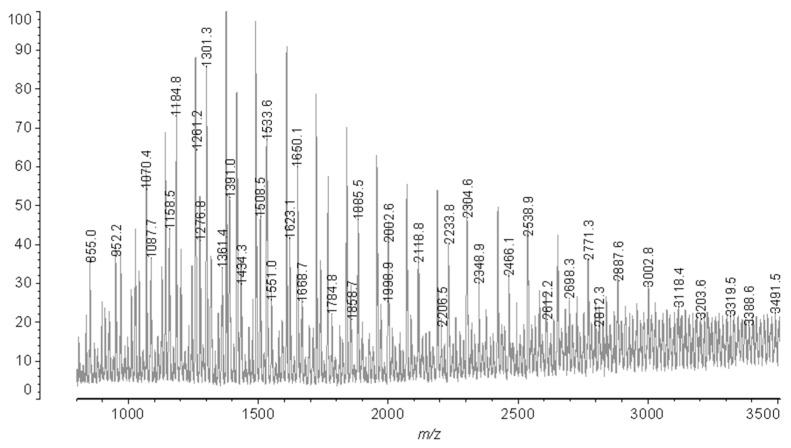
MALDI-TOF spectrum of PIPGE/PBO/PIPGE-diol (18).

Employing MALDI-TOF mass spectrometry for the analysis of copolymers allows us to obtain additional data about the structure of macromolecules formed during the process. Figure 12 presents, for example, the MALDI-TOF spectrum of PIPGE/PBO/PIPGE-diol (18).

Several signals revealed in the spectrum represent macromolecules containing a block of PBO and two blocks of PIPGE, which possess a central fragment –OC_3_H_6_OC_3_H_6_O– derived from the initiator and two end OH groups. They form adducts with sodium ions. Characterization data of this copolymer involving the main macromolecules at m/z 1301.3 to 4802.9 are shown in Table 4. Signals of the highest intensities (>40%) represent macromolecules containing 3–29 mers of BO and 7–28 mers of IPGE. Macromolecules (1–12) possessing 3, 4, 7, 14, 17, and 20 mers of BO contain ≥50 mol % of IPGE mers. On the other hand, macromolecules with 21, 22, and 26–29 mers of BO contain ≤50 mol % of IPGE mers. The average ratio of BO/IPGE is 40/60.

**Figure 13:**
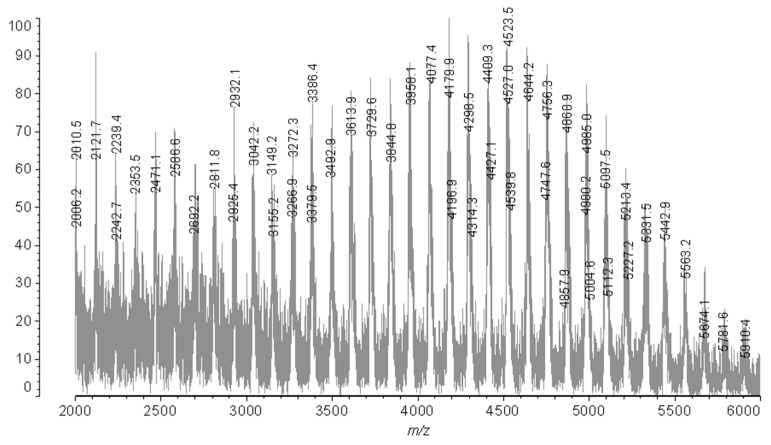
MALDI-TOF spectrum of PIPGE/PAGE/PIPGE-diol (19).

Another example is PIPGE/PAGE/PIPGE-diol (19), for which the MALDI-TOF spectrum is shown in Figure 13.

Several signals in the spectrum represent macromolecules that form adducts with sodium ions. Characterization data of this copolymer, involving the main macromolecules at m/z 2932.1 to 5220.4, are presented in Table 5. Signals of the highest intensity (>40%) represent macromolecules with 6–27 mers of AGE and 14–23 mers of IPGE. Macromolecules (1–7) possessing 6, 9, 12, or 15–17 mers of AGE contain >50 mol % of IPGE mers, whereas macromolecules with 18, 20, 21, 23, 24, or 27 mers of AGE contain <50 mol % of IPGE mers. The average ratio of AGE/IPGE is 50/50 in this case.

### 3.3. Random Copolyether-Diols

**Scheme 6:**
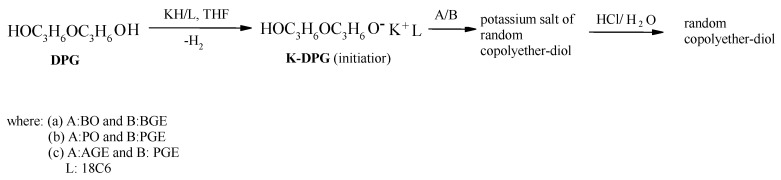
Preparation of initiator and random copolyether-diols by ring-opening copolymerization of monosubstituted oxiranes.

Additional, three new random copolyether-diols were synthesized in this study by using monopotassium salt of dipropylene glycol as an initiator, which was activated by macrocyclic ligand 18-crown-6 (K-DPG/L). In all systems, the initial concentration of each monomer was 2.0 and the initial concentration of each initiator was 0.1 mol/dm^3^. Two monosubstituted oxiranes as comonomers were mixed and added to the initiator solution in tetrahydrofuran. After several hours at room temperature, this results in copolyether-diols after protonation (Scheme 6).

The products obtained were analyzed by several methods. The SEC method indicated that all copolymers are unimodal. Their characterization is shown in Table 6.

In general, the ^13^C NMR spectra of copolymers reveal strong signals of carbons present in polymer chains (CH_2_ and CH) and substituents, as well as weak signals in carbons’ terminal groups (Table 7).

Weak signals of CH_3_ carbons derived from initiator were observed in the ^13^C NMR spectrum at 17.3 ppm of copolymers (21) and (23). The lack of signals from the terminal carbon of the CH(CH_3_)OH group from the initiator at 65.0 and 67.1 ppm indicated that propagation occurs in two directions. This is possible due to cation exchange reaction. In the spectrum of copolymer (22), carbon signals of the allyl group (at 117.0 and 136.0 ppm) were not found, which indicated that the chain transfer reaction to PO did not occur. However, in the spectrum of copolymer (23), weak signals of *cis*-propenyloxy groups were shown (at 101.0 and 146.0 ppm), resulting from isomerization of the allyloxy groups in the substituent.

**Figure 14:**
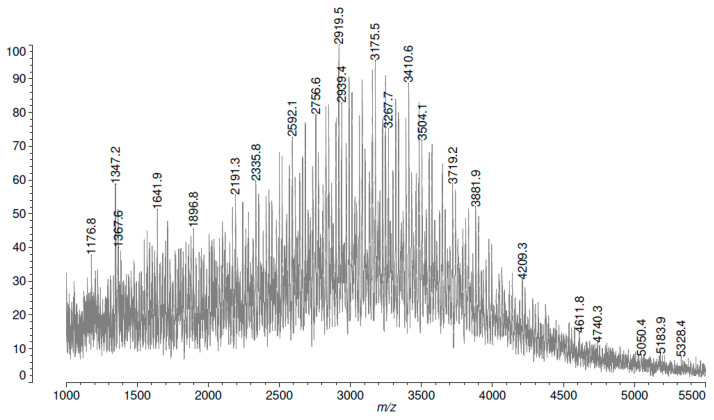
MALDI-TOF spectrum of BO/BGE copolyether-diol (21).

Analysis of copolymers by MALDI-TOF mass spectrometry provided additional data concerning their structure. The spectrum of the BO/BGE copolymer is shown in Figure 14. 

Several signals were observed in the spectrum at m/z 1500 to 4000. They represent macromolecules containing central fragment –OC_3_H_6_OC_3_H_6_O– derived from the initiator, two end H atoms, and various numbers of mers of both monomers. The composition of copolyether-diols that form adducts with sodium ions was determined and is presented in Table 8. Macromolecules containing 9–14 mers of BO and 9–16 mers of BGE were identified with an average BO/BGE ratio of 1/1. Signals of homopolymers, i.e., PBO-diols and PBGE-diols, were not found in the spectrum.

**Figure 15:**
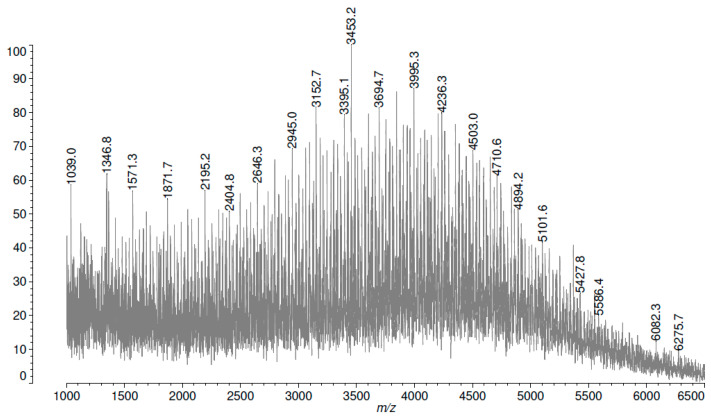
MALDI-TOF spectrum of PO/PGE copolyether-diol (22).

Figure 15 presents the MALDI-TOF spectrum of the PO/PGE copolymer.

The spectrum of this copolymer reveals several peaks in the range of m/z 1000 to 5500. The composition of copolyether-diols that form adducts with sodium ions was determined (Table 9). Macromolecules containing 2–8 mers of PO and 16–28 mers of PGE were identified. In all macromolecules, the PO/PGE ratio is about 1/4. This means that PO indicates the tendency to homopolymerization. Indeed, several signals of PPO-diols were identified in the spectrum (Table 10). Homopolymers of PO contain a relatively high number of mers, i.e., in the range 45–81.

**Figure 16:**
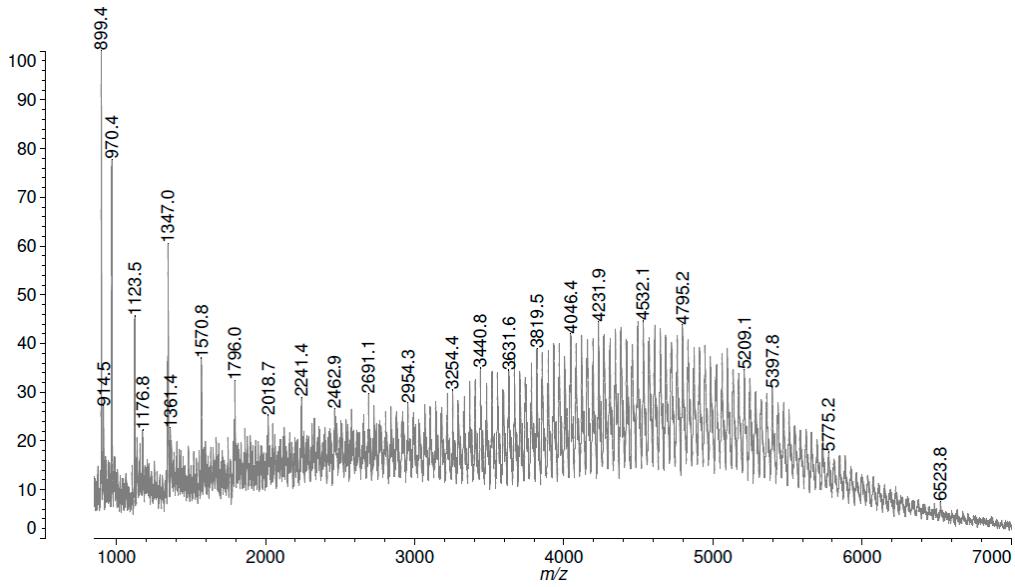
MALDI-TOF spectrum of AGE/PGE copolyether-diol (23).

Figure 16 shows the MALDI-TOF spectrum of the AGE/PGE copolymer.

Several signals were present in the spectrum in the range of m/z 1000 to 6000 (Table 11). They represent macromolecules of copolyether-diols containing 7 or 12–14 mers of AGE and 4–24 mers of PGE were identified. For example, macromolecules containing 14 mers of AGE contain 5–23 mers of PGE. Analysis of the spectrum also indicated signals that represent homopolymers, i.e., PAGE-diols and PPGE-diols (Table 12 and Table 13).

All the results derived from the MALDI-TOF MS analysis confirmed the presence of a copolymeric structure in the studied random copolyether-diols, with a lack of homopolymeric diol macromolecules for BO/BGE, and some of them for the PO/PGE and AGE/PGE systems.

## 4. Conclusions

The application of monopotassium dipropylene glycoxide activated 18C6 for anionic ring-opening polymerization of monosubstituted oxiranes allowed us to prepare several kinds of new macrodiols. These are homopolyether-diols and copolyether-diols, i.e., triblock and random ones, which were characterized by SEC, ^13^C NMR, MALDI-TOF, and FTIR techniques. The main conclusions are as follows:

- It is possible to initiate polymerizations of the discussed oxiranes in mild homogeneous conditions, i.e., in THF solution at room temperature and normal pressure.
- The growth of polymer chains occurs in two directions due to cation exchange reaction.
- Synthesized macrodiols are unimodal and have M_n_ ≈ M_calc_ (1500–8300) and low dispersity (M_w_/M_n_ = 1.08–1.18).
- Monopotassium salts of homopolyether-diols, i.e., PPO-diol, PBO-diol, and PAGE-diol, appeared to be useful macroinitiators for the preparation of new triblock copolyether-diols from glycidyl ethers.
- In BO/BGE, random copolymerization initiated with K-DPG/18C6 macromolecules of copolyether-diol formed exclusively.
- Macromolecules of copolyether-diol and also homopolyether PPO-diol were identified in the PO/PGE system.
- AGE and PGE comonomers reacted to create random copolyether-diol as well as homopolymers, i.e., PAGE-diol and PPGE-diol.
- Several prepared homopolymer-diols and copolyether-diols could be useful for the fabrication of new thermoplastic or crosslinked polyurethanes.

## Figures and Tables

**Table 1:** Characterization of polyether-diols prepared with K-DPG activated by 18C6 as an initiator in THF at room temperature; [I]_o_ = [18C6]_o_ = 0.1 mol/dm^3^.

No.	Monomer	[M]_o_ mol/dm^3^	Yield %	Reaction Time, h	M_calc_^a/^	M_n_ (SEC)	M_w_/M_n_ (SEC)	Initiator Efficiency, *f* ^b/^
1	PO	2.0	99	95	1161	1500	1.09	0.77
2	PO	5.0	98	230	2903	3200	1.12	0.91
3	PO	10.0	99	400	5805	6000	1.13	0.96
4	BO	2.0	98	170	1442	1900	1.10	-
5	BO	5.0	99	300	3605	4100	1.11	-
6	BO	9.0	97	510	6489	6700	1.14	-
7	AGE	2.0	99	45	2283	2700	1.14	-
8	AGE	5.0	98	110	5708	5800	1.13	-
9	IPGE	2.0	99	50	2322	2500	1.08	-
10	IPGE	5.0	97	120	5805	5900	1.09	-
11	PGE	2.0	99	60	3000	3500	1.13	-
12	PGE	5.0	96	140	7500	7600	1.14	-
13	BGE	2.0	99	80	3283	3600	1.15	-
14	BGE	5.0	97	190	8208	8300	1.16	-

^a/^ M_calc_ = ([M]_o_/[I]_o_)M_mon_. ^b/^ f = M_calc_/M_n_ (SEC).

**Table 2:** Characterization data of triblock copolyether-diols by size exclusion chromatography (SEC) chromatography.

No.	Copolymer	Yield %	Reaction Time, h	Retention Time, min		M_calc_^a/^	M_n_ SEC	M_w_/M_n_ SEC
				Macroinitiator	Copolymer			
15	PAGE/PPO/PAGE	99.4	150	7.60	6.90	3578	3400	1.16
16	PPGE/PPO/PPGE	99.2	160	7.60	6.81	4299	4000	1.09
17	PAGE/PBO/PAGE	99.5	225	7.21	6.59	3859	3600	1.18
18	PIPGE/PBO/PIPGE	99.0	220	7.21	6.61	3899	3700	1.17
19	PIPGE/PAGE/PIPGE	99.3	110	6.18	5.90	4740	4500	1.13
20	PBGE/PAGE/PBGE	99.1	145	6.18	5.62	5700	5200	1.10

^a/^ M_calc_ = ([A]_o_/[I]_o_)M_A_ + ([B]_o_/[I]_o_)M_B_ + M_DPG_.

**Table 3:** Content of *cis*-propenyloxy groups derived from isomerization of allyloxy groups in copolyether-diols.

No.	Copolymer	OCH_2_CH=CH_2_ (mol %)	OCH=CHCH_3_ (mol %)
15	PAGE/PPO/PAGE	99.6	0.2
17	PAGE/PBO/PAGE	94.9	5.1
19	PIPGE/PAGE/PIPGE	97.5	2.5
20	PBGE/PAGE/PBGE	97.2	2.8

**Table 4:** Composition of PIPGE/PBO/PIPGE triblock copolyether-diol (18) determined by MALDI-TOF mass spectrometry.

No.	Formula ^a^	Intensity of Signal (>40%)	m/z (MALDI-TOF)	m/z (calc)	BO/IPGE (mol %/mol %)
1	IPGE_4_BO_3_IPGE_4_	82.3	1301.3	1302.8	27/73
2	IPGE_5_BO_3_IPGE_5_	47.7	1535.6	1535.1	23/77
3	IPGE_6_BO_3_IPGE_5_	76.3	1650.1	1651.3	21/79
4	IPGE_8_BO_3_IPGE_8_	81.7	2233.8	2232.1	16/84
5	IPGE_8_BO_4_IPGE_8_	40.2	2304.6	2304.1	20/80
6	IPGE_14_BO_4_IPGE_13_	56.4	3580.0	3581.8	13/87
7	IPGE_4_BO_7_IPGE_3_	100	1360.4	1358.9	50/50
8	IPGE_14_BO_7_IPGE_14_	50.8	3798.7	3798.3	20/80
9	IPGE_13_BO_14_IPGE_12_	64.3	4069.0	4069.7	36/64
10	IPGE_11_BO_17_IPGE_10_	42.9	3823.8	3822.4	45/55
11	IPGE_12_BO_17_IPGE_11_	43.5	4055.3	4054.7	42/58
12	IPGE_11_BO_20_IPGE_10_	40.1	4037.8	4038.7	49/51
13	IPGE_8_BO_21_IPGE_8_	41.8	3530.0	3530.0	57/43
14	IPGE_10_BO_21_IPGE_9_	57.0	3879.8	3878.5	53/47
15	IPGE_11_BO_22_IPGE_11_	44.1	4299.7	4299.1	50/50
16	IPGE_11_BO_26_IPGE_10_	51.1	4470.5	4471.4	55/45
17	IPGE_8_BO_27_IPGE_7_	47.4	3846.0	3846.5	64/36
18	IPGE_10_BO_28_IPGE_10_	45.4	4500.1	4499.5	58/42
19	IPGE_9_BO_29_IPGE_8_	40.3	4223.5	4223.1	63/37
20	IPGE_11_BO_29_IPGE_11_	51.7	4802.9	4803.9	57/43

^a^ The formula does not involve the fragment –OC_3_H_6_OC_3_H_6_O–, derived from the initiator as well as two end H atoms.

**Table 5:** Composition of PIPGE/PAGE/PIPGE triblock copolyether-diol (19) determined by MALDI-TOF mass spectrometry.

No.	Formula ^a^	Intensity of the Main Signal (>40%)	m/z (exp)	m/z (calc)	AGE/IPGE (mol %/mol %)
1	IPGE_9_AGE_6_IPGE_9_	49.3	2932.1	2932.9	25/75
2	IPGE_12_AGE_5_IPGE_11_	74.4	3856.5	3856.1	28/72
3	IPGE_9_AGE_12_IPGE_9_	43.9	3618.3	3617.7	40/60
4	IPGE_10_AGE_12_IPGE_9_	42.1	3734.0	3733.9	39/61
5	IPGE_10_AGE_15_IPGE_9_	100	4077.4	4076.3	44/56
6	IPGE_9_AGE_16_IPGE_8_	42.0	3958.1	3958.1	49/51
7	IPGE_10_AGE_17_IPGE_9_	68.1	4304.4	4304.6	47/53
8	IPGE_8_AGE_18_IPGE_7_	59.6	3954.4	3953.9	55/45
9	IPGE_7_AGE_20_IPGE_7_	93.5	4065.5	4065.9	60/40
10	IPGE_9_AGE_21_IPGE_9_	76.7	4644.2	4644.7	54/46
11	IPGE_8_AGE_23_IPGE_7_	69.6	4523.5	4524.4	60/40
12	IPGE_11_AGE_23_IPGE_10_	45.7	5220.4	5221.4	52/48
13	IPGE_9_AGE_24_IPGE_8_	71.7	4869.0	4870.9	60/40
14	IPGE_7_AGE_27_IPGE_6_	88.1	4747.6	4748.7	68/32
15	IPGE_8_AGE_27_IPGE_8_	40.1	5097.5	5097.7	63/37

^a^ The formula does not involve the fragment –OC_3_H_6_OC_3_H_6_O–, derived from the initiator as well as two end H atoms.

**Table 6:** Characterization of copolyether-diols by SEC technique.

No.	Copolymer	Yield (%)	Reaction Time, h	M_calc_^a^	M_n_ (SEC)	M_w_/M_n_ (SEC)
21	BO/BGE	99.7	250	4850.4	4500	1.11
22	PO/PGE	98.9	165	4299.2	4100	1.15
23	AGE/PGE	99.2	120	5420.4	5100	1.18

^a^ M_calc_ = ([A]_o_/[I]_o_)M_A_ + ([B]_o_/[I]_o_)M_B_ + M_DPG._

**Table 7:** ^13^C NMR chemical shifts (in ppm) of terminal carbons present in copolyether-diols.

No.	Copolymer	δ_C__H(R)OH_ (Chain)
21	BO/BGE	73.46 and 73.70 69.36
22	PO/PGE	65.55 and 67.10 69.42
23	AGE/PGE	68.62 and 69.1 69.42

**Table 8:** Composition of BO/BGE random copolyether-diol (21) determined by MALDI-TOF mass spectrometry.

No.	Formula ^a^	Number of BGE Mers (*n*) and Intensities of the Main Signals (>40%) ^b^	Signals of Highest Intensity (>40%)		
			m/z (exp)	m/z (calc)	BO/BGE (mol %/mol %)
1	BO_9_BGE_n_	9 (49.1),13, 15, 16	2191.3	2193.3	50/50
2	BO_10_BGE_n_	5, 10, 11 (47.6)	2683.7	2685.2	48/52
		14 (83.3)	3176.5	3177.1	42/58
		15 (70.2)	3338.6	3340.7	40/60
3	BO_11_BGE_n_	8, 12 (100)	2919.5	2920.9	48/5
		14 (86.5)	3248.8	3249.3	44/56
		15 (52.0)	3412.6	3413.5	43/57
		16 (45.2)	3574.9	3577.0	40/60
4	BO_12_BGE_n_	12 (78.0)	2991.0	2993.0	50/50
		14, 15 (66.0)	3484.1	3485.6	44/56
5	BO_13_BGE_n_	12 (67.7)	3064.5	3065.1	52/48
		13 (62.6)	3228.3	3229.3	50/50
6	BO_14_BGE_n_	11 (40.1)	2974.5	2973.0	56/44

^a^ The formula does not involve the fragment –OC_3_H_6_OC_3_H_6_O–, derived from the initiator as well as two end H atoms. ^b^ The intensities of other signals are 10–40%.

**Table 9:** Composition of PO/PGE random copolyether–diol (22) determined by MALDI-TOF mass spectrometry.

No.	Formula	Number of PGE Mers (*n*) and Intensities of the Main Signals (>40%)	Signals of Highest Intensity (>40%)		
			m/z (exp)	m/z (calc)	PO/PGE (mol %/mol %)
1	PO_2_PGE_n_	22 (55.3), 23, 24	3578.3	3577.1	8/92
		26, 27			
2	PO_3_PGE_n_	16, 19 (78.4)	3185.2	3184.6	14/86
		24(48.9)	3936.1	3935.5	11/89
		25(42.2)	4086.2	4085.7	11/89
		26, 27(43.9)	4386.5	4386.0	10/90
3	PO_4_PGE_n_	19, 20 (92.7)	3393.1	3392.0	17/83
		23 (60.1)	3844.4	3843.4	15/85
		24 (60.9)	3995.3	3993.6	14/86
		25 (69.1)	4144.3	4143.7	14/86
		27 (58.5), 28	4445.4	4444.1	13/86
4	PO_5_PGE_n_	18 (55.4), 19	3152.7	3150.7	21/79
		20 (100), 21	3453.2	3451.0	20/80
		23 (87.2)	3902.3	3901.5	18/82
		24 (80.9), 25	4052.1	4051.7	17/83
		26 (87.8)	4352.7	4352.0	16/84
		27 (78.5)	4503.0	4502.2	16/84
		28 (56.2)	4652.5	4662.3	15/84
5	PO_6_PGE_n_	16, 20, 21, 22			
		24, 25 (70.4)	4260.5	4259.7	19/81
		26, 27 (66.7)	4559.9	4560.4	18/82
		28 (60.6)	4710.6	4710.2	16/84
6	PO_7_PGE_n_	19, 21, 22, 24, 28			
7	PO_8_PGE_n_	24, 25			

**Table 10:** PPO-diols accompanied by PO/PGE copolymerization initiated with K-DPG/L.

No.	Formula	Intensities of the Main Signals (>10%)	m/z (exp)	m/z (calc)
1	PO_45_	34.8	2772.4	2770.9
2	PO_47_	42.0	2886.9	2887.0
3	PO_49_	48.1	3003.1	3003.2
4	PO_50_	69.4	3061.9	3061.3
5	PO_51_	19.2	3119.8	3119.4
6	PO_76_	27.2	4570.7	4571.4
7	PO_79_	58.2	4744.6	4745.6
8	PO_81_	30.7	4860.4	4861.8

**Table 11:** Composition of AGE/PGE random copolyether–diol determined by MALDI-TOF mass spectrometry.

No.	Formula	Number of PGE Mers (*n*) and Intensities of the Main Signals (>40%)	Signals of Highest Intensity (>40%)		
			m/z (exp)	m/z (calc)	AGE/PGE (mol %/mol %)
1	AGE_7_PGE_n_	7, 13, 14, 15, 16, 17, 18 (80.3)	3668.9	3669.3	28/72
		19, 20(72.7), 21, 23	3969.2	3969.6	26/74
		24(78.3)	4571.0	4570.2	22/78
2	AGE_12_PGE_n_	4, 7, 9, 14, 15 (64.5)	3780.1	3779.4	44/56
		16, 17, 18 (54.8)	4230.9	4229.9	40/60
		19 (100)	4381.5	4381.5	39/61
		20 (96.6)	4532.1	4530.3	38/62
		21, 22, 23(58.8)	4981.7	4980.8	37/63
		24 (60.6)	5130.1	5130.9	36/64
3	AGE_13_PGE_n_	4, 6, 7, 9, 18, 20			
		21(41.2), 22, 23, 24	4795.2	4794.6	38/62
4	AGE_14_PGE_n_	5,6, 7, 11, 15, 16			
		18 (77.2)	4459.1	4458.2	44/56
		20 (68.6), 21	4759.5	4758.5	41/59
		22 (50.0)	5059.6	5058.9	39/61
		23 (50.6)	5209.1	5209.0	38/62

**Table 12:** PAGE-diols accompanied by AGE/PGE copolymerization initiated with K-DPG/L.

No.	Formula	Intensities of the Main Signals (>10%)	m/z (MALDI-TOF)	m/z (calc)
1	AGE_34_	10.1	4039.7	4038.9
2	AGE_35_	16.7	4153.8	4153.1
3	AGE_36_	10.5	4268.5	4267.2
4	AGE_37_	99.9	4382.5	4381.4
5	AGE_38_	89.9	4496.2	4495.5
6	AGE_39_	67.8	4610.2	4609.6
7	AGE_40_	13.1	4722.5	4723.8

**Table 13:** PPGE-diols accompanied AGE/PGE copolymerization initiated with K-DPG/L.

No.	Formula	Intensities of the Main Signals (>10%)	m/z (MALDI-TOF)	m/z (calc)
1	PGE_27_	12.2	4212.2	4211.8
2	PGE_28_	15.2	4362.0	4361.9
3	PGE_29_	16.1	4511.3	4512.1
4	PGE_32_	10.2	4961.1	4962.6
5	PGE_33_	10.7	5111.5	5112.8
6	PGE_34_	14.6	5261.0	5262.9
